# Quinton Deeley

**DOI:** 10.1192/bjb.2022.51

**Published:** 2022-12

**Authors:** Abdi Sanati

**Figure:**
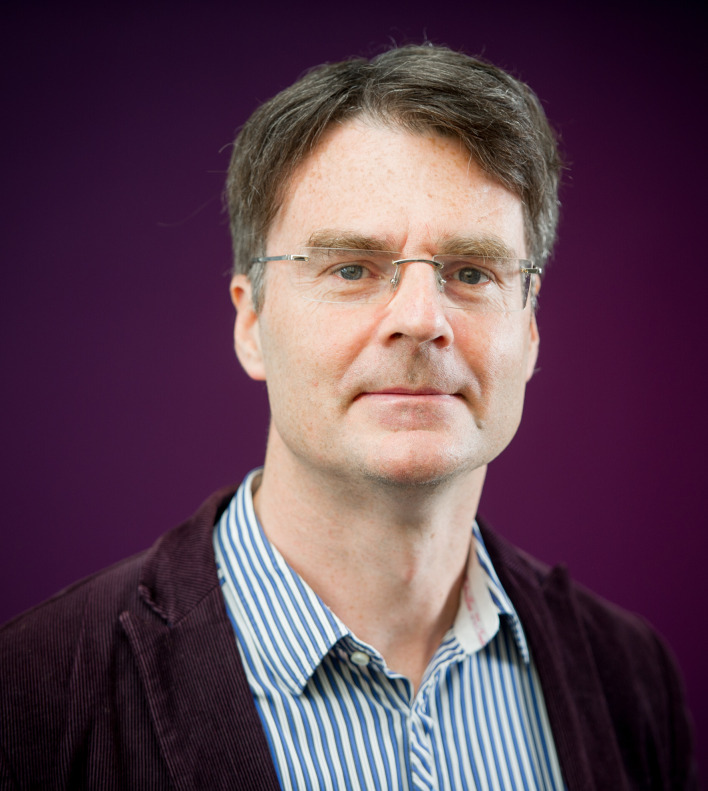

Dr Deeley is an honorary consultant neuropsychiatrist in the National Autism Unit at Bethlem Royal Hospital and in the Neuropsychiatry Brain Injury Clinic at the Maudsley Hospital, two hospitals within South London and Maudsley NHS Foundation Trust. He is also a senior lecturer in forensic and neurodevelopmental sciences at the Institute of Psychiatry, Psychology & Neuroscience (IOPPN), King's College London. He chairs the Cultural and Social Neuroscience Research Group at the IOPPN and the Maudsley Philosophy Group.

**Thank you very much for agreeing to be interviewed, Dr Deeley. How do you see the status of services for people with autism in the UK?**

Well, I think these services continue to be in a state of development. There has been a trend in recent years towards the setting up of dedicated teams for working with people with ASD [autism spectrum disorder] – for diagnosis, to support community mental health teams and also to do some in-reach work. But that particular model is somewhat unevenly implemented across the UK. There continue to be some specialist in-patient services for people with ASD, some of which are low secure, some of which are locked wards and some of which are open wards. But again, they are geographically very unevenly distributed. So we have a situation where some areas are relatively well served. But for many parts of the UK, if there is a need for in-patient admission to a specialist service it might require being out of area and sometimes very many miles from home. The other way of thinking about autism services is to think about developing the skills of generic services to work with people on the autistic spectrum. In general terms that must be the most important approach to meeting the mental health needs of people on the autistic spectrum because it's a common condition affecting up to 1% of the adult population and also the child population, based on well-conducted epidemiological studies. It's a common condition and highly associated with mental health conditions. So many people on the autistic spectrum will be presenting to primary and secondary and specialist healthcare services across the course of their life. Consequently, I think the key to improving mental health services for these people will be improving awareness of autism across the entire NHS.

**Thank you. Do you think that psychiatrists need to have specific training in autism?**

Yes, I would say that psychiatrists do need to have specific training in autism. This is partly, of course, in terms of the basic knowledge base – for example, the diagnostic criteria for an autistic spectrum condition. But I think it goes beyond that. It also is important for people to develop the skills and the clinical sense of knowing how to assess somebody on the autistic spectrum, how to manage differential diagnosis and the detection of coexisting mental health conditions, given the very high rates of comorbidity. There should also be training about the problem of diagnostic overshadowing – that is to say that sometimes it can be difficult to distinguish an autistic trait from a mental health symptom – for example, distinguishing between, say, a preference for routine or repetitive behaviours and an obsession or compulsion. This can be achieved through courses, but also, I think, through increasing the skills of existing mental health teams so that clinicians in training can benefit from learning about people with ASD under supervision. So ultimately, it goes back to postgraduate medical education and the supervision of trainees.

**You mentioned diagnostic overshadowing. I was thinking of this in terms of some of philosophical issues raised by autism. One way we look at psychosis is the alteration of reality. And autistic people already have a different approach to reality, they see reality differently. How does that alteration manifest in autism? Do we expect the same alteration that we see in non-autistic people in autistic people or would psychosis have a totally different meaning in autism?**

Well, in order to diagnose a psychotic disorder, one has to be able to describe the characteristic features of psychosis, whether that's hallucinations or delusions. It may be harder to detect these symptoms in some people with ASD because of difficulties they have describing their internal world or in terms of perspective-taking, understanding what they may be taken to mean by somebody other than themselves. That can lead to clinical misinterpretation, seeing psychotic symptoms when they are not present. So, for example, a person with ASD may have a preoccupying interest or idea that seems unusual to somebody else, and when this is expressed under conditions of stress it could be misinterpreted as representing, for example, a delusional preoccupation. Also, when people with ASD become acutely stressed this can exacerbate problems with communication and expressing connections between ideas, which could be misinterpreted as schizophrenic thought disorder. Equally, people with ASD may present with a reduced range of facial expressions, muted non-verbal communication and executive difficulties, which can be mistaken as a negative schizophrenic syndrome if you lack a history of the person. Obviously, reduced non-verbal communication will be more consistent in its presentation – it's part of an ASD – whereas it would fluctuate with episodes of illness more in the case of psychosis. Psychosis can be misdiagnosed in people with ASD for all of these reasons.

However, the fundamental question you're raising is how the senses of reality that people with ASD have differ from those of people with psychosis. Psychosis really involves a breakdown in ‘reality testing’ – our connection with reality. And this can be manifested through delusions or through abnormal perceptions. Abnormal perceptions are, of course, not part of the core characteristics of ASD. So, if hallucinations are present that's more consistent with a psychotic process than ASD. Probably the hardest area to judge, and which I think you alluded to in your question, is the differentiation of delusions from the special interests or preoccupations or, indeed, interpretations of reality of people with ASD. This is where a careful assessment of the individual and their overall context is essential. So, for example, many people with ASD, because of years of social rejection, exclusion, mistreatment, exploitation, develop a mistrustful interpretive style. They develop a very low threshold for interpreting the intentions of others as hostile – in other words, paranoid personality traits which in the absence of a careful history can be potentially misinterpreted as expressions of paranoid delusions. Sometimes it can be difficult to tell the difference between the two. I think in terms of the oddity of the preoccupations of people with ASD, there should still be a qualitative difference between the bizarreness or inherent implausibility of delusional ideas and the preoccupations of people with ASD. People with ASD may have unusual interests and preoccupations and may misinterpret social interactions, but they shouldn't have the systematic, bizarre or unbelievable quality of the delusions of a person with psychosis.

**What do you think of the way autistic people perceive time?**

I think it's likely that people with ASD do not perceive time in a single way. The experience of time is complex and variable for all people, whether they have autism or not, it depends upon circumstances and context. The sense of time fluctuates. People with ASD have a preference for routine, sameness or predictability that can be associated with an intolerance of uncertainty. And so under those circumstances – for example, if a person with ASD is awaiting something that is anxiety-provoking, such as a change in their living circumstances, like moving from home into a community placement – the heightened sense of anxiety can produce a very burdensome sense of the passage of time. The uncertainty and the lack of transition from one certain state to another certain state draws out time and alters its perceived quality so that it becomes very distressing. Equally, people with ASD may become very absorbed in certain activities, particularly activities of special interest. And of course, during states of profound absorption the sense of time alters – it seems to pass more quickly. So I think the kind of variations in the sense of time that people without ASD have will also be present in people with ASD, but they'll often be intensified in people with ASD.

**I wanted to move close to the legal aspects. Now, capacity is important and I was wondering how, in your opinion, does the concept of capacity and the way that is defined in law address the complexities of decision-making in autistic people?**

Well, again, I think one has to take each case as it comes but I do agree that the concept of capacity and the assessment of capacity can pose special challenges in people with ASD. So, for example, people with ASD who are in the normal intelligence range – that may include people who are highly intelligent – are capable of reasoning at a very high level about certain topics. This can confront you with a situation where you have somebody who is able to understand and retain much information which is pertinent to a particular decision, but where the particular difficulty relates to weighing information in the balance in order to arrive at a decision. And I think that is often where judgements about capacity can be very difficult. A person may have islands of insight or high levels of understanding about a particular decision. For example, if we take the case of somebody who has a health condition who's not engaging with normal investigation or treatment, there may be many aspects of the health condition that they research and understand well, but nevertheless, they may have a very rigid thinking style and form very strong opinions which they find difficult to change in light of relevant information. Problems with perspective-taking may also make it harder to take into account alternative points of view about what may be in their best interests or beneficial to them. And so they may discount alternative courses of action. A reluctance to have certain physical investigations can have a sensory basis, it may be that the person becomes anxious about a certain type of invasive investigation. But equally, a refusal to accept a particular investigation or treatment may result from problems with consequential thinking, problems of flexibly thinking through the consequences of not having a certain investigation or treatment. So the Mental Capacity Act can be applied to people on the autistic spectrum. But often the decision-making difficulties can be subtle and require careful weighing up because in certain respects there may be evidence which would generally be taken to suggest the presence of capacity, but there may be elements of reasoning – the weight placed on certain information – which indicate that capacity is not present. Of course, if in doubt, then the cases can always be referred up to the Court of Protection. I'm not aware of a study which looks at the proportion of cases in the Court of Protection where people are on the autistic spectrum, but it would be an interesting study to do because I suspect that many complex cases will relate to capacity decisions in people on the autistic spectrum.

**From capacity we can move to best interests. If someone lacks capacity by virtue of their autism, it's very hard to imagine them without the autism and know what they would decide if they were capacitous. That could pose a challenge.**

It does pose a challenge. The treatment plan that's being proposed or the decision that's being proposed for a person with autism should always take into account their autism and their preferences to the extent that that's possible. So even if a decision is being made which goes against the wishes of a person with autism, it remains important to think through how that decision can be implemented in a way which is takes into account, as much as possible, that person's particular sensibility and their preferences. So, for example, if it was a case of, say, a decision to support the person to have a medical investigation against their will or to be discharged into supported living arrangements against their will, the likelihood of those interventions being successful will be increased by taking into account the particular pattern of preferences of that person. So, for example, with the medical investigation, you know, how could the sensory aspects of the investigation be mitigated? Who can accompany the person to reassure them the most? When do they need to be told about the decision, to reduce anxiety? What language should be used to explain the decision to them? And so on. So, I think decisions should always be attentive to a person's autistic characteristics, even if it's something which is made in their best interests on the basis that they lack capacity with respect to that decision.

**I wanted to ask what you think of the concept of neurodiversity that sees autism as some form of diversity in the way that the person presents in society.**

I think the concept of neurodiversity is an important innovation because it recognises that there is diversity within the human population and that people with autistic traits or characteristics are an important part of the human population. In actual fact, many people on the autistic spectrum do not have a need for care over and above that of the general population. And also many have special talents, abilities or aptitudes which are useful either to themselves or to other people or indeed society as a whole. For example, the biographies of many successful scientists suggest the presence of autistic traits or indeed autism. At the present time there are very successful individuals in financial services and the internet industry who self-identify as or are known to be on the autistic spectrum. So all of this would be consistent with the idea that there is variation in the population and that autistic traits lead to many individual characteristics which are conducive to creativity and technical and scientific innovation. That would include, for example, the propensity to form intense interests, an interest in systems, taxonomy and causal explanation, unusual memory skills and heightened perceptual skills in some cases. Nor should we underestimate the potentially liberating consequences for scientific thinking or intellectual innovation of not being preoccupied with thinking about other people, with social cognition. If your cognition is tilted or oriented more towards thinking about systems rather than people, then that means you can devote more time and cognitive resources to problem-solving and understanding science and all of these factors are likely conducive to the association between autistic traits and scientific creativity. It is very important to recognise that there are many people who would meet criteria for autism with respect to their traits, who for the most part are not distressed or socially affected to an extent that requires support. They are able to function very productively within their particular context.

**And finally, what advice do you have for young psychiatrists who want to increase their skills in autism?**

Well, for a good starting point, there is the Royal College of Psychiatrists’ report, *CR228 The Psychiatric Management of Autism in Adults.* That's an up-to-date document. It's an overview that gives a good knowledge base. But for a psychiatrist who wants to become a developmental psychiatrist, one way is to specialise in mental health in learning [intellectual] disability – because there, of course, you have a very high level of exposure to neurodevelopmental disorders. For the general adult psychiatrist I would recommend seeking out special interest sessions with clinicians within their organisation who have a special interest in neurodevelopmental conditions – ASD, but also ADHD. It's always useful to learn about both these conditions, not least because they're highly associated.

**Thank you very much. It was great talking to you.**

